# Population Genetic Structure and Diversity of Cryptic Species of the Plant Genus *Macrocarpaea* (Gentianaceae) from the Tropical Andes

**DOI:** 10.3390/plants12081710

**Published:** 2023-04-20

**Authors:** Julien C. Vieu, Darina Koubínová, Jason R. Grant

**Affiliations:** Institute of Biology, University of Neuchâtel, Rue Emile-Argand 11, 2000 Neuchâtel, Switzerland

**Keywords:** AFLP, *Macrocarpaea*, cryptic species, phylogeography, Pleistocene climatic oscillations, refugia

## Abstract

The Pleistocene climatic oscillations (PCO) that provoked several cycles of glacial–interglacial periods are thought to have profoundly affected species distribution, richness and diversity around the world. While the effect of the PCO on population dynamics at temperate latitudes is well known, considerable questions remain about its impact on the biodiversity of neotropical mountains. Here, we use amplified fragment length polymorphism molecular markers (AFLPs) to investigate the phylogeography and genetic structure of 13 plant species belonging to the gentian genus *Macrocarpaea* (Gentianaceae) in the tropical Andes. These woody herbs, shrubs or small trees show complex and potentially reticulated relationships, including cryptic species. We show that populations of *M. xerantifulva* in the dry system of the Rio Marañón in northern Peru have lower levels of genetic diversity compared to other sampled species. We suggest that this is due to a recent demographic bottleneck resulting from the contraction of the montane wet forests into refugia because of the expansion of the dry system into the valley during the glacial cycles of the PCO. This may imply that the ecosystems of different valleys of the Andes might have responded differently to the PCO.

## 1. Introduction

Mountain ranges are often considered as speciation centers that significantly contribute to enhancing species diversity on Earth [1,2]. They are thought to favor speciation through their high overall physiographic heterogeneity that facilitates both allopatric divergence between populations occurring on different montane systems and adaptive divergence along elevational gradients within the montane systems [3,4]. Numerous studies have documented rapid species divergences (radiations) of taxa taking place almost immediately after the colonization of a particular montane region [5,6,7,8]. While the evidence for the contribution of adaptive divergence remains insignificant [9], it is clear that allopatric speciation plays a determinant role in driving many montane radiations [2,10].

Climate change has also been proposed as a potentially important driver of speciation in montane regions, particularly in non-vagile organisms such as plants [11,12]. It is relatively well justified that the Pleistocene climatic oscillations (PCO, 2.58-0.01 mya) have profoundly affected the demographic evolution of montane elements by repeatedly provoking species movement on the elevational gradients: descending during the glacial periods and ascending during interglacial periods [11]. At temperate latitudes, the glacial periods of the PCO are generally considered as a time of range expansion of montane cold-adapted taxa and their secondary contact in the lowlands [11,13,14]; however, examples of divergence or at least isolation in peripheral refugia valleys have also been documented [15,16]. In contrast, the effect of the PCO glacial–interglacial cycles on neotropical montane biodiversity is still understudied.

Among neotropical mountains, the tropical Andes are of particular interest because they have exceptional levels of diversity and endemism [17]. It has been proposed that the PCO significantly contributed to the overall diversity found in the region [18,19]; however, their potential consequences currently remain poorly understood. The dramatic altitudinal lowering of the upper forest line (UFL, currently at 1800 m a.s.l.) during glacial periods is well documented by the palynological record sampled across the tropical Andes. However, it shows considerable interregional and often also an intraregional variation [20,21]. It is less known whether the PCO affected the level of precipitation in the tropical Andes and the Neotropics in general [22]. On one hand, the “refugia model” suggests that glacial periods were characterized by increased aridity in the Neotropics [23,24]. According to this theory, during glacial periods, montane forest populations were compressed into refugia as a consequence of both the lowering of the UFL, resulting from cooling, and the expansion of dry conditions on montane foothills [22]. Under this scenario, it is expected that the action of genetic drift in small populations separated into distinct refugia would result in an overall loss of genetic diversity within the refugia, but also a potential genetic differentiation between the refugia. Populations from different refugia that accumulate new mutations would likely show reciprocal monophyly, and the gene flow between isolated populations from different refugia would be limited [22]. The periods of rapid re-expansion of the populations during the interglacial periods could have also induced a gradient of loss of genetic diversity between the source populations in the refugia and the expansion front. On the other hand, the opponent “moist forest” model considers that the PCO did not change the overall precipitation and the glacial periods remained relatively wet [25,26]. This scenario does not predict compression at the foothills during the glacial periods, but instead a migration of montane elements down into the lowlands, resulting in extensive secondary contact between populations, followed by subsequent upslope migration and re-fragmentation during the interglacial periods. In this case, the PCO should not induce a significant loss of genetic diversity and the patterns of the geographical genetic structure are expected to be more complex. Genetic diversity would be preserved throughout the species range by habitat heterogeneity and significant gene flow between populations [22].

Palynological evidence has shown that the Amazon-facing slopes of the eastern Andean cordilleras remained fairly wet during the glacial periods of the PCO [25,27]. However, it is less known whether the montane forests that occur in the inter-Andean valleys, in the proximity of the areas where seasonally dry forests occur nowadays, suffered more extensively from compressions induced by the potential intensification of drought during the glacial periods of the PCO. In addition, according to the “moist forests” model, the potential secondary contacts initiated through the downslope migration of the populations in the wet Amazon-facing slopes of the eastern cordilleras might have represented a challenge for the maintenance of incipient species integrity if the populations differentiating in allopatry did not have enough time to complete reproductive isolation. To better understand the potential complex impact of the PCO on the neotropical montane vegetation, more phylogeographic studies are needed.

Here, we used amplified fragment length polymorphism genetic markers (AFLPs) to examine the phylogeography and population genetics of plant species belonging to the genus *Macrocarpaea* (Gentianaceae). The genus is restricted to the mountains of the Neotropics (the Andes, the Guayana Highlands, Mesoamerica, the Greater Antilles, and the Atlantic rainforest) [28]. Currently, there are 118 recognized species [28,29,30,31,32,33,34,35,36,37,38,39,40,41,42]. Here, we specifically examined 13 species. Most of them are cryptic, i.e., morphologically similar but genetically different, and endemic to the lower montane forests (LMF < 1800 m a.s.l.) of Ecuador and northern Peru, whereas many other species of the genus occur in middle-elevation montane forests of the tropical Andes (MMF, 1000–3500 m), or upper montane forests (UMF > 1800 m; [43]. Previous studies strongly supported this group of *Macrocarpaea* species as monophyletic [43,44,45]. The age of the most recent common ancestor (MRCA) of the group has been dated at 2 mya with all subsequent divergence events in the group occurring within the period of the PCO. We specifically focus on *M. xerantifulva* and *M. claireae* since previously conflicting results were obtained by different approaches, with one of them contradicting the morphological and other traits (i.e., both species being sister, as expected, *versus*, both species nesting in separate clades) [43,45]. *Macrocarpaea claireae* J. R. Grant is an unbranched shrub or small tree 1.5–2.0 m tall, with a 2 cm diam. trunk; wood hollow in trunk and in branches; stems terete to quadrangular, 6–8 mm diam.; leaves oval, elliptic to ovate, and petiolate. It is found around Valladolid in Zamora-Chinchipe province in southern Ecuador [33]. *M. xerantifulva* J. R. Grant is found in Cajamarca and Piura provinces in northern Peru. It has similar characteristics to *M. claireae* but is notable in herbarium specimens in having stems that dry yellowish gold in color (*vs.* green), and calyces that dry brownish (*vs.* drying black) [28,31].

Similarly, despite other processes such as the impact of interspecific interactions, the genetic background of the above-mentioned species may be influenced by species dispersal distance, sexual reproduction *vs.* vegetative spread, individual lifespan and climatic fit of the species. Here, we specifically focus on the impact of the PCO because, in previous studies [43,45], the splits between the species occurred during the PCO, which was not determined as the main influence but its contribution could not be neglected based on previous studies. We use the AFLP approach as it is convenient to detect polymorphism in DNA when no information about the genome is available.

Here, we (1) explore and compare the spatial genetic structure of these species with the use of the AFLP technique; (2) test whether the genetic diversity of populations of the closely related *M. claireae* and *M. xerantifulva* is lower than in the other species, as expected if the “refugia model” was more plausible and the internal Andean valleys were dryer during the PCO, or not (thus potentially supporting the “moist forest” model); and (3) detect potential genetic (distance-based) variability among the previously morphologically determined lineages as a basis and indication for further studies.

## 2. Results

### 2.1. Population Structure

**Figure 1:**
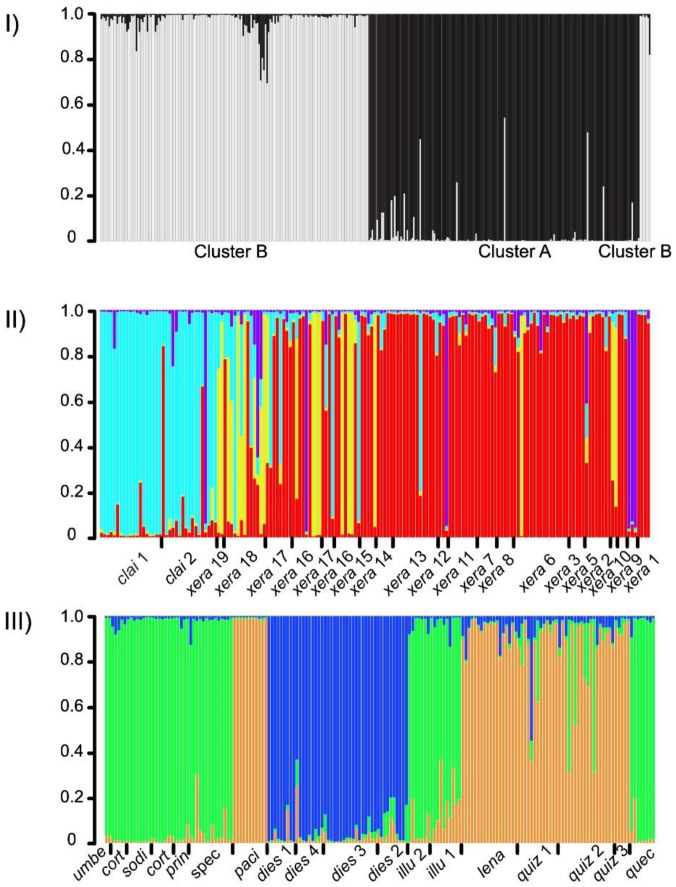
Bar plots showing the average cluster assignment probability in nested STRUCTURE analyses of the AFLP data for individuals from different species of *Macrocarpaea*. Each bar represents one individual, and the color indicates the source of the genetic cluster of its genotype. Bars are sorted according to decreasing latitude (north–south gradient). (**I**) Analyses including all the individuals sampled in the study. (**II**) Analyses performed on the individuals assigned to cluster A, representing *M. claireae* and *M. xerantifulva*. (**III**) Analyses performed on the individual assigned to cluster B, representing all the ten remaining species. The abbreviations of the names of the populations (see Table 1) are indicated below the bars and are formed by the first 4 characters of the species name.

The ∆*K* statistic of the STRUCTURE analyses performed on all the genotypes of all specimens sampled in the study supported an optimal value of *K* = 2 (Figure S1A). Here, we denominate the clusters as A and B, with cluster A (black) being composed of populations of *M. claireae* and *M. xerantifulva* and cluster B (white) being composed of the populations of all remaining species (see Material and Methods). Levels of admixture between these two clusters were generally very low (Figure 1I). Contrary to ∆*K*, estimates of mean log-likelihood (mean L(*K*)) increased steadily after *K* = 2 before reaching a plateau at *K* = 8. This discrepancy between the two statistics indicates that the ∆*K* statistic identified the highest level of hierarchical structure in our dataset but that additional layers of the genetic structure are likely present inside the clusters.

**Figure 2:**
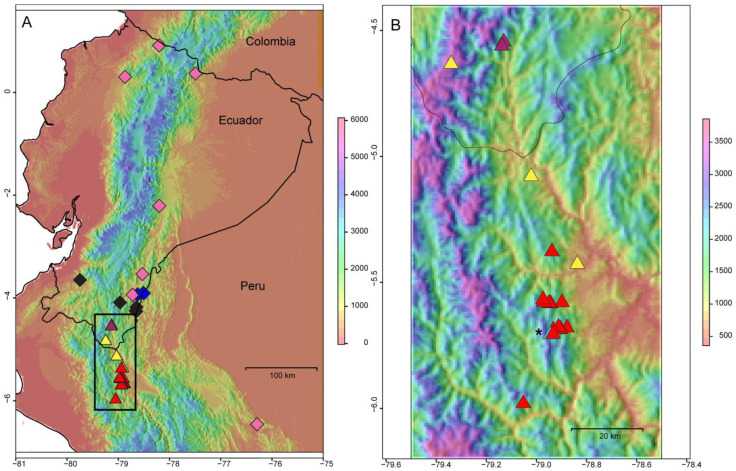
Map of the sampling sites of the populations analyzed in this study. (**A**) All populations sampled. Diamonds indicate the populations of the genetic cluster B inferred with the Bayesian clustering program STRUCTURE. The pink diamond corresponds to the populations of the sub-cluster B1 (*M. cortinae, M. illuminata, M. lenae, M. pringleana, M. quechua, M. sodiroana, M.* sp. nov., *M. umbellata*; see Table 1), the black to the sub-cluster B2 (*M. pacifica*, *lenae*, *quizhpei*) and the dark blue to the sub-cluster B3 (*M. dies-viridis*). Triangles indicate the populations of cluster A. The magenta triangles correspond to the sub-cluster A1 (*M. claireae*), the yellow to A2 and the red to A3 (*M. xerantifulva*). (**B**) Zoom on the Rio Chinchipe valley and the distribution of cluster A (*M. claireae* and *xerantifulva*), including the sub-cluster A4 (light blue and asterisk) not visible in (**A**).

In the subsequent analyses performed inside cluster B, both ∆*K* and mean L(*K*) favored *K* = 4 (Figure S1B). The bar plot shows the probability of assignment of individuals to a particular genetic cluster, with the individuals sorted according to increasing distance from the northernmost individual (north: left, south: right, Figure 2B and Figure 1II). Two genetic groups are dominant. One, labeled as A1 and shown as sky blue in the north, is highly dominant in the populations of *M. claireae* 1 and *M. claireae* 2, while A3 (red) is found as dominant among the central and southern populations. In between, cluster A2 (yellow) is often found in central populations, showing mixed genetic information with A1 and A3. Its prevalence tends to decrease toward the south and it is absent from the two northernmost populations (A1). The populations with the highest prevalence of A2 are *M. xerantifulva* 19 and *M. xerantifulva* 2 but both are formed by only two individuals. Finally, the A4 (violet) cluster is relatively rare but is present along the entire geographic distribution. The only population where all individuals show this characteristic is *M. xerantifulva* 9. Overall, the level of admixture in individuals is low, except in Peruvian populations close to the border with Ecuador (i.e., “central populations”). Inside cluster B, the ∆*K* statistic favored *K* = 3 while the mean L(*K*) only reached a plateau for *K* = 6 (Figure S1C). This indicates that several layers of the genetic structure might remain inside the identified clusters. All populations of *M. dies-viridis* fall in cluster B3 (blue). The cluster B1 (green) regroups populations from eight different species and covers a very large geographic distribution (Figure 2A and Figure 1III). The level of admixture in individuals is very low except for a few individuals from the three populations of *M. quizhpei*.

### 2.2. Descriptive Statistics of Diversity

Gene diversity (*H_j_*, see Table 1) is almost two times higher in the populations of cluster B (mean *H_j_* = 0.150_;_ sd = 0.025) than in those of cluster A (mean *H_j_* = 0.089; sd = 0.026) and the difference in means between the two clusters is highly significant (*T*_(34_._5)_ = 7.076, *p* < 0.001). The results of the hierarchical AMOVAs performed either by grouping populations by species or by their prevailing assignment with Bayesian clustering (Table 2) revealed that, in both cases, more than half of the total genetic variation (54.88% and 57.07%, respectively) can be explained by the variation between individuals inside the populations. In both designs, as expected based on the clusters being formed by several species, the proportion of the variation explained by differences among groups (species or cluster) is two times higher than the variation explained by the difference among populations within groups. This indicates that the groups’ boundaries explain a substantial proportion of the genetic variation. However, the proportion of the variation explained by the species (33.06%) is higher than the proportion of the variation explained by STRUCTURE genetic clusters (27.25%), suggesting that the species; boundaries better capture the overall genetic variation. In both designs, all types of *F* statistics tested with permutations were highly significant (Table 2). The fact that F_SC_ statistics (variation among sub-groups divided by the sum of variation among sub-groups and variation within sub-groups) are significant indicates that populations inside the groups are still appreciably differentiated.

**Figure 3:**
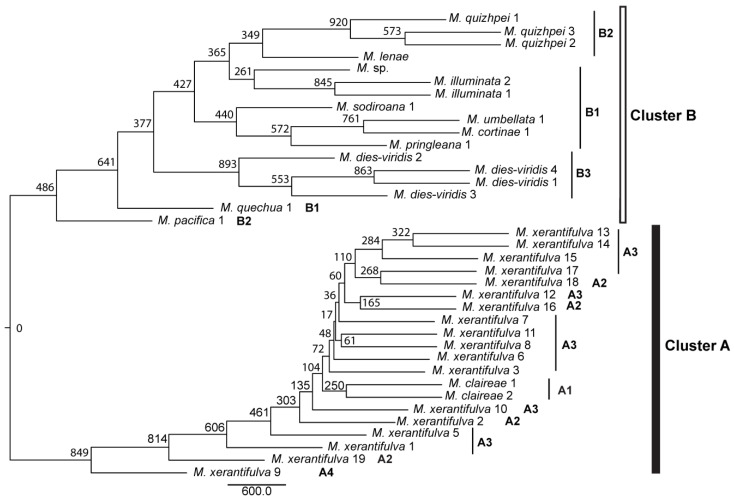
Neighbor-joining (NJ) dendrogram computed from Nei’s genetic distances from AFLP loci. Values at the nodes correspond to bootstrap support (N/1000) obtained by recalculating genetic distances from bootstrapped loci. The codes A, A1 to A4, B and B1 to B3 refer to genetic clusters identified by the Bayesian clustering method performed with STRUCTURE.

The NJ dendrogram computed from Nei’s genetic distance (Figure 3) displays the same basal dichotomy as inferred with STRUCTURE. Cluster A has relatively high bootstrap support (849/1000) and the population *M. xerantifulva* 9, which is the only population predominantly assigned to the sub-cluster A4, is well supported as being well differentiated within the cluster. Then, inside cluster A, populations do not group according to their STRUCTURE assignment. Similarly, internodes are short and bootstrap support is low, indicating potentially little divergence among populations. Inside cluster B, the bootstrap support is in general higher and the internodes are much longer than in cluster A, very likely indicating a larger divergence between populations inside cluster B. The only sub-cluster identified by STRUCTURE that is strongly supported as monophyletic is cluster B3. *M. dies-viridis* (893/1000), *M. illuminata* (845/1000) and *M. quizhpei* are strongly supported as monophyletic (920/1000).

### 2.3. Spatial Genetic Structure

**Figure 4:**
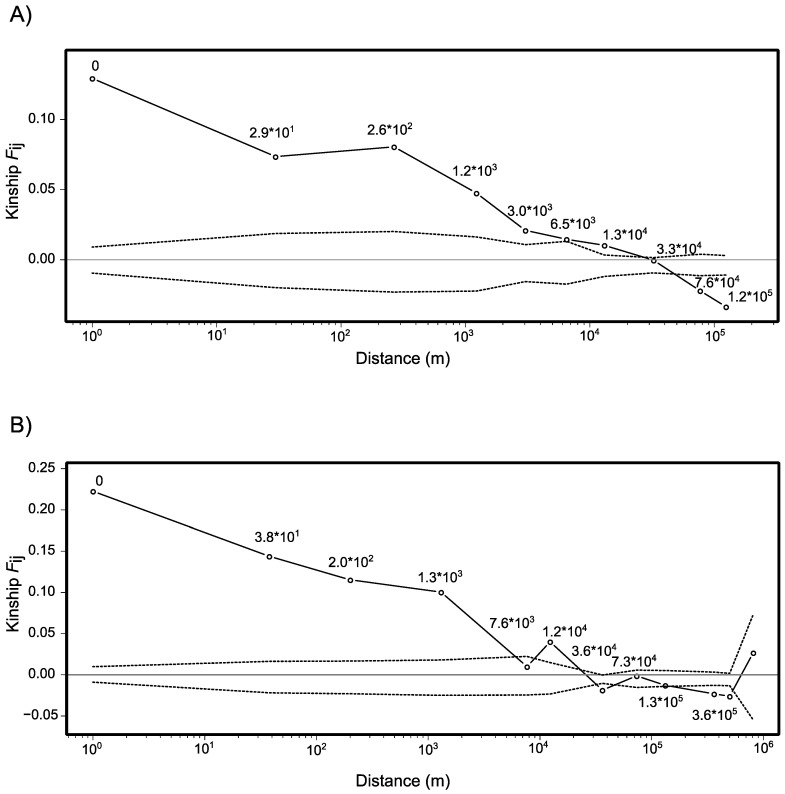
Correlograms of kinship coefficients (*Fij*) per distance class represented on a logarithm scale; (**A**) for cluster A identified by STRUCTURE; (**B**) for cluster B. The plain line represents the value measured on the data. The dotted line represents the confidence interval of the permutations and indicates the null expectation of no spatial genetic structure. The circles show the mean distance estimated inside a particular distance class (see Methods). Closed circles indicate values significantly different from the null expectation while open circles correspond to the null expectation.

The correlograms showing the spatial autocorrelation of kinship (*F_ij_*), measured for cluster A (Figure 4A) and cluster B (Figure 4B) independently, are similar in the way they reveal significantly higher kinship than expected by chance across a relatively large distance. For cluster A, the kinship becomes null only between 6.5 and 33 km. For cluster B, the kinship becomes null between approximately 6 and 36 km. The slope of the regression of kinship against ln of distance is significantly more negative than expected under a scenario of the absence of spatial genetic structure both for cluster A (*b*_F_ = −0.0147; *p* < 0.01) and cluster B (*b*_F_ = −0.0148; *p* < 0.01). The two clusters have relatively similar Sp statistics with *Sp* = 0.0158 and *Sp* = 0.0172 for cluster A and cluster B, respectively. Finally, it should be noted that the intra-individual kinship (*F*_(0)_), which corresponds to the *F*_IT_ when individuals from different populations are compared [46], is much higher for the cluster B (*F*_(0)_ = 0.22) than for the cluster A (*F*_(0)_ = 0.13). This indicates that the sum of inbreeding inside the populations and the overall differentiation between populations is higher in group B. As gene diversity is higher in the populations of cluster B (see above and Table 2), the difference is likely a consequence of higher differentiation between populations in cluster B rather than higher inbreeding.

## 3. Discussion

Here, we analyzed a dataset of 203 loci of AFLP markers from 345 individuals of 13 closely related plant species belonging to the genus *Macrocarpaea* (Gentianaceae): *M. claireae*, *M. cortinae*, *M. dies-viridis*, *M. illuminata*, *M. lenae*, *M. pacifica*, *M. pringleana*, *M. quechua*, *M. quizhpei*, *M. sodiroana*, *M*. sp. nov., *M. umbellata*, and *M. xerantifulva*. We performed STRUCTURE and NJ analyses to examine the genetic structure of the populations and to shed light on the effect of the PCO on the biodiversity of neotropical mountains.

### 3.1. Deep Genetic Structure

The unrooted NJ dendrogram constructed from matrices of genetic distances between populations and the Bayesian clustering analyses performed at the individual level (STRUCTURE) identified the same basal dichotomy in the genetic structure of the group of species we studied. Cluster B is formed by species with populations occurring on the Amazon-facing slopes of the eastern cordilleras from northern Ecuador to northern Peru, plus the unique population of *M. pacifica* that occurs on the Pacific-facing slopes of the western cordillera in southern Ecuador and the unique populations of *M. sodiroana* and *M. umbellata* that occur on the Pacific facing slopes of the western cordillera in northern Ecuador. Cluster A comprises the two populations of *M. claireae in* southern Ecuador and the 18 sampled populations of *M. xerantifulva* from the Rio Chinchipe valley in Peru. The low bootstrap of populations detected is probably a result of the fact that most of the populations belong to the same species and have very small samples.

Interestingly, one of the rare studies that addressed the phylogeography of a montane plant group from the same region identified the Rio Chinchipe valley as a source population of *Ceroxylon echinulatum* Galeano (Arecaceae) for the colonization of the eastern and western cordilleras of the Andes followed by subsequent migration toward the north during the Quaternary [47]. Here, instead, we propose that cluster A likely remained separated in this valley, and the low level of admixture detected between clusters A and B indicates that they have evolved in relative isolation (spatial and/or genetic) from one another. Naturally, this isolation theory needs to be supported by a closer inspection of the valleys surrounding the Rio Chinchipe valley, but it should be noted that, despite several visits on the eastern side of the Rio Chinchipe valley (populations were sampled on the western side of the river), we were not able to find populations from cluster A, while other *Macrocarpaea* species from different clades were collected at a higher elevation. Visits further south, on the other side of the Rio Marañón, were also unfruitful.

### 3.2. The Concept of Species Inside the Species Complex

Several elements from our results raise important questions regarding the definition of the species boundaries inside this *Macrocarpaea* species complex. Notably, neither the genetic distance-based dendrogram nor the Bayesian clustering analyses support the species boundary defined based on morphological data and field observations for *M. claireae* and *M. xerantifulva*. *Macrocarpaea claireae* in cluster A is relatively deeply nested in the middle of the populations of *M. xerantifulva* on the dendrogram. The Bayesian clustering analyses reveal that *M. claireae* 1 and the northernmost population of *M. claireae* 2, which were sampled a few kilometers apart from one another (<3 km), are members of the same genetic cluster (cluster A1). The fact that individuals belonging to cluster A1 are also found mixed with other genetic clusters in other populations close to the border between Ecuador and Peru indicates that *M. claireae* and *M. xerantifulva* may be the same species that displays a geographic genetic structure. The relatively low branch length at the internodes for clade A on the dendrogram also suggests relatively low genetic differentiation between populations inside the cluster, which support the hypothesis of the presence of a single species inside cluster A. However, as the samples of the populations of cluster A are very small, the structure detected within this cluster is probably not sufficiently strong from a statistical point of view and the species boundaries need to be tested with more solid approaches. Thus, future studies should revisit the taxonomic status of *M. claireae* and *M. xerantifulva*.

A population genetic approach like we employ in this study has great potential to first detect the genetic boundaries of cryptic species and then to assist the taxonomic assignment of individuals to one species or another. Surprisingly, the ∆*K* statistics [48] only favored 3 genetic groups inside cluster B where we recognized 10 taxonomic species [28,35]. We think that this small number of groups identified is very likely the outcome of the detection of a deep genetic structure inside cluster B, rather than the presence of a maximum of three differentiated genetic groups [49]. This is illustrated by the low level of admixture detected inside each sub-cluster (Figure 1III). In comparison, the mean log-likelihood for the number of clusters (mean L(*K*); Figure S1C) only started to reach a plateau around *K* = 6 and thus indicates the potential presence of additional levels of genetic structure. The comparison of the partitioning of the genetic variance (AMOVA, Table 2), either assuming the species boundaries or the genetic clusters as the highest level of genetic structure, revealed that the species boundaries explained a greater proportion of the genetic variance, which also supports the idea that the genetic clusters identified by STRUCTURE did not capture the totality of the genetic structure present in our dataset. 

### 3.3. Differences in Gene Diversity and the Dry Refugia

We found a significant deficit of about 40% in gene diversity in the populations of *M. xerantifulva* (mean *H_j_* = 0.089) in comparison to the populations from cluster B (mean *H_j_* = 0.150). Compared with estimates in the literature for other perennial and predominantly outcrossing plants [50], gene diversity is relatively low for cluster B and very low for *M. xerantifulva*. The very low levels of gene diversity found in *M. xerantifulva* indicate that this species very likely went through a demographic bottleneck, which fits the prediction of the dry refugia model [22]. This, in turn, suggests that the Rio Chinchipe valley might have been drier during the cold cycles of the PCO than nowadays and that the populations of *M. xerantifulva* re-colonized the valley only recently. An alternative hypothesis to explain the low genetic diversity in *M. xerantifulva* could be that it has recently been founded by a small number of individuals (founder event; [51]). This hypothesis is quickly refuted by the fact that the basal divergence between *M. xerantifulva* and cluster B likely dates back to their common ancestor some 2 Myr [45]. According to the prediction of the dry refugia hypothesis, it should be possible to locate the position of the refugium by identifying the population or the region that has the highest level of gene diversity. In *M. xerantifulva* there is no clear candidate population that differs from the others. The only exception is *M. xerantifulva* 12, but it is a very small population (N = 3, *H_j_* = 0.132) which limits its significance. Another approach to identifying refugia could consist of assessing the diversity of genetic clusters inside populations (genotype diversity). Using this approach, *M. xerantifulva* 18, which is in northern Peru in the proximity to the border with Ecuador, and *M. xerantifulva* 18, which is located about 20 km further south, are the best candidates. Considering that if an intensification of drought occurred in the Rio Chinchipe valley, it should have been more severe in the south, the position of refugia in that part of the valley is not illogical. Nevertheless, this pattern of genotype richness cannot be taken as the ultimate evidence for the position of refugia. Secondary contact zones can also generate a hotspot of genotype diversity (genetic cline) and both scenarios are relatively difficult to set apart [52].

### 3.4. Spatial Genetic Structure

Analyses of the spatial genetic structure (SGS) revealed a strikingly similar pattern of SGS between *M. xerantifulva* and genetic cluster B. Both groups show significantly high kinship over a relatively long distance (6–30 km) and thus have a relatively weak SGS. The regression slopes of the relationship between pairwise kinships and pairwise logarithms of distances (*b*_F_) were significantly more negative than expected by chance, which indicates a significant effect of isolation by distance. However, this last assertion should be taken with caution as the maximal distances we considered were very large in comparison with other studies (124 km for *M. xerantifulva* and 847 km for cluster B). Therefore, it is not surprising to detect an effect of isolation by distance at such a large spatial scale. In addition, despite their similarity, the SGS estimates for cluster B are likely to be meaningless as this group is potentially composed of several species while SGS is usually estimated at the intraspecific level [53]. Consequently, we limit the interpretation of SGS to *M. xerantifulva*. The significantly high kinship we found at a relatively large distance in *M. xerantifulva* suggests that the populations of this species are relatively well connected genetically. *Macrocarpaea* species are usually predominantly pollinated by nectar-feeding bats [28,54] which have a foraging radius of 30 to 50 km and thus can disperse pollen across considerable distances [55]. It is therefore possible that the high population connectivity we detect is enhanced by bat pollination. Finally, the *Sp* statistic for *M. xerantifulva* (*Sp* = 0.0158) is extremely similar to the rare estimate that exists in the literature for bat-pollinated plant species [56,57].

### 3.5. Limitations and Alternative Explanations

We recognize that our study and the interpretation of the results have limitations and that there may be alternative explanations to our observations and conclusions. One of the limitations is the uneven sampling due to the extremely difficult accessibility and possibilities of finding of the populations and sample size. For example, some of the populations in cluster A, compared to cluster B, include only a few representatives, which might have biased the result showing low genetic variance, i.e., a low genetic difference observed among few samples may be low by chance and would be different if more individuals were sampled. However, the mean sampling size per population in cluster A is 8.5 samples/population and in cluster B it is 10.3. This difference is not substantial. Moreover, in the cases where only a few individuals were sampled, the populations were also small, i.e., all or the majority of the detected individuals at the given locality were sampled. Such small populations usually indicate that the genetic variability would be low due to the effects of limited gene flow and inbreeding [58,59]. In addition, some of the individuals were more clustered together at the sampling localities (e.g., *M. claireae* 2), while some were more distant from each other both horizontally and vertically (to a maximum of 500 m as mentioned in Materials and Methods), which is the case of, e.g., *M. claireae* 1 where the samples were found at altitudes in the range of 100 m. These discrepancies, sometimes caused by the biology of the respective species and sometimes by the conditions at the locality, might have also affected the observed genetic diversity within and between populations.

The results shown in the dendrogram in this study are different to those found in previous molecular studies on *Macrocarpaea* [43,45]. These discrepancies are probably due to the molecular markers used (AFLP versus ribosomal and plastid loci), a different sampling pattern (different number of individuals and different species included) and missing data in one of the species showing different positions in this study and in previous publications (e.g., *M. claireae* analyzed previously was missing plastid data which might have affected the analyses, as for other data both plastid and ribosomal markers were used, see Appendix S1 in [45]. In the main phylogeny (Figure 2) in this publication [45], *M. claireae* was sister to *M. quizhpei*, while in Appendix S3, it was sister to *M. xerantifulva*. Conflicts between plastid, mitochondrial, ribosomal and nuclear data are commonly reported; also, AFLP data are not particularly convenient for phylogenetic reconstruction. The figure presented here is a dendrogram, not a phylogenetic tree; however, the results presented here support the sister relationship of *M. claireae* and *M. xerantifulva*, which also corresponds to their morphology and other biological traits. As previously mentioned, more extensive sampling and application of different methods are needed for more precise phylogenetic conclusions.

## 4. Materials and Methods

### 4.1. Sampling

**Figure 5:**
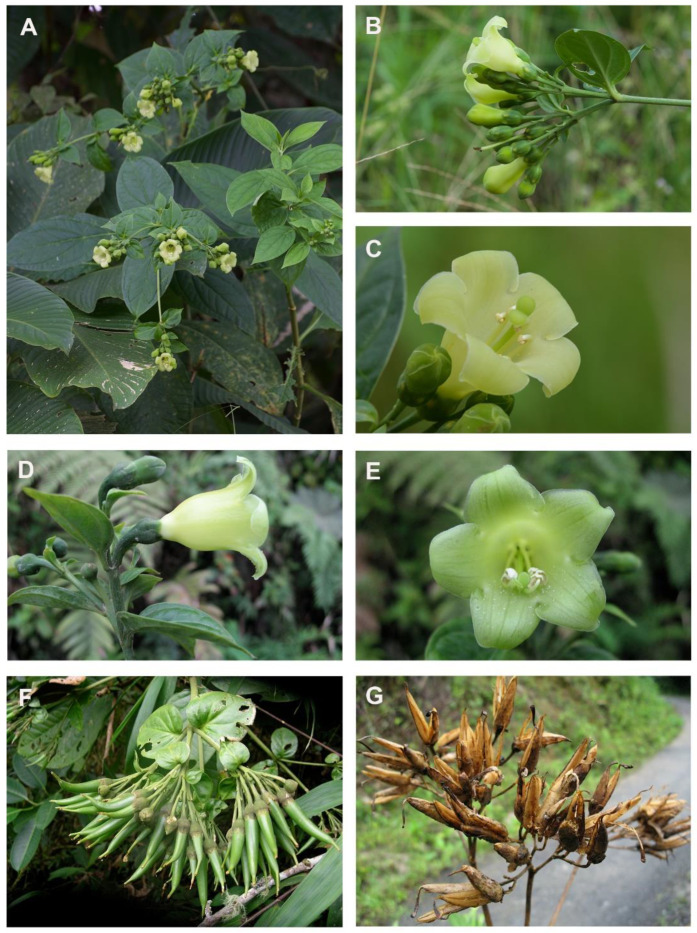
Morphological diversity in *Macrocarpaea* (Gentianaceae). (**A**–**C**) *M. cortinae* J.R. Grant; (**D**,**E**) *M. claireae* J.R. Grant; (**F**) *M. dies-viridis* J.R. Grant; (**G**) *Macrocarpaea* sp.

Samples of 13 *Macrocarpaea* species were collected between 2011 and 2013 at 37 localities in nine regions of Ecuador and Peru (Figure 2, Table 1). Eleven of the species are small cryptic trees (3–6 m), with minor differences in their morphology (Figure 5; [28,35]. Three species (*M. pacifica* J.R. Grant, *M. sodiroana* Gilg, and *M. umbellata* Weaver & J.R. Grant) occur on Pacific-facing slopes of the Andes, while the other ten occur on Amazon-facing slopes of the eastern cordilleras in the Amotape-Huancabamba (AHZ) zone in southern Ecuador and northern Peru (*M. claireae* J.R. Grant, *M. cortinae* J.R. Grant, *M. dies-viridis* J.R. Grant, *M. illuminata* J.R. Grant, *M. lenae* J.R. Grant, *M. pringleana* J.R. Grant, *M. quechua* J.R. Grant, *M. quizhpei* J.R. Grant, *M*. sp. nov. and *M. xerantifulva* J.R. Grant; Figure 5). Specifically, *M. xerantifulva* J.R. Grant is composed of herbaceous/woody herbs (1–3 m) in northern Peru between the Cordillera Real in the west and the Cordillera del Condor in the east, in the Rio Chinchipe valley (Figure 2). This valley is connected in the south to the main Rio Marañón valley, which is characterized by the vegetation of seasonally dry tropical forests (SDTF). Since *Macrocarpaea* species generally occur in relatively small, sparse populations that are often difficult to locate, it was not possible to follow a predefined sampling design. Most species were sampled from one to a few and sometimes small populations. The exception is *M. xerantifulva*, that was sampled more intensively from 18 populations throughout its distribution range. Since no preliminary information was available about the population structure for any species in the genus, we arbitrarily decided that plants from patches separated by at least 500 m belonged to different populations. Some species are branched from the base; therefore, distant but random sampling ensured the collection of genetically different individuals. With its large, night-blooming, yellow-to-greenish-colored bell-shaped flowers that produce large quantities of nectar, *Macrocarpaea* is geared towards nocturnal bat pollination. These primary pollinators facilitate outcrossing across scattered populations. The nectar is also appreciated by other nocturnal visitors such as moths, as well as early diurnal visitors such as hummingbirds and butterflies that arrive before the corollas fall.

### 4.2. DNA Extraction and Genotyping

Total genomic DNA was extracted from silica gel-dried leaf samples with standard cetyltrimethylammonium bromide (CTAB)-chloroform extraction followed by isopropanol precipitation and ethanol washing [60]. The DNA samples were eluted in water and purified for a second time using the DNeasy plant mini kit (Qiagen Ltd., Crawley, UK), following the Mini protocol and starting at step 11 (i.e., after the lysis part of the protocol). DNA quality was checked by electrophoreses on agarose gels and DNA concentrations were measured with a Nanodrop 2000 spectrophotometer (Thermo Scientific, Wilmington, DE, USA). DNA concentrations were standardized to 10–20 ηg.μL^−1^.

Amplified fragment length polymorphism markers (AFLPs) were generated for 345 individuals and 103 duplicates (29.7% of the dataset), following the protocol of Vos et al. [61] with the modifications of Parisod & Christin [62]. *EcoRI* and *MseI* enzymes (Bioconcept, Allschwil, Switzerland) were used for digestion. All cycles of digestion-ligation were performed on the same thermocycler machine (VWR, Radnor, PA, USA). After a preliminary test on 24 selective primers combinations, three were retained based on their levels of polymorphism and reproducibility: M-CGA/E-AGC, M-CCA/E-ACA and M-CCG/E-ATC, labelled with the fluorescent dyes FAM, VIC and NED (Applied Biosystems, Foster City, CA, USA), respectively. Amplified PCR products were pooled with GeneScan 500 LIZ Size Standard (Applied Biosystems, Foster City, CA, USA), sent to Macrogen Inc. (Seoul, Republic of Korea) and separated on the ABI 3730XLs DNA Analyser (Applied Biosystems, Foster City, CA, USA). Fragment sizes were estimated with GeneMapper v4.0 (Applied Biosystems, Foster City, CA, USA) using the AFLP default peak detection parameters. Marker bins were set manually for each primer combination and only fragments within the range of 50–500 bp with a peak height of a minimum of 50 relative fluorescent units (rfu) were considered for generating a marker (bin). Peak scoring on the bin sets was performed automatically by the software and resulting tables with peak heights and sizes were exported and processed with scanAFLP v. 1.3 [63] using the default threshold options and following the steps (2) and (3) described in Hermann et al. [63]. This script assesses presence/absence within a particular locus, relying on locus-specific fluorescent signal intensity thresholds estimated from qualities of the fragment signal intensity distribution (0 for absence and 1 for presence of a band at a particular position). For each primer combination, we exported the binary data matrix, referred to as MatrixB in Hermann et al. [63], that we assembled in a single binary matrix containing a total of 522 loci. At this step, loci present in fewer than 10 individuals and more than all the individuals minus 10 were discarded. The matrix with 306 remaining loci was used to estimate the locus-specific error rate on duplicated samples by applying the mismatch approach of Bonin et al. [64]. Every locus with an error rate larger than 10% was removed from the binary matrix, resulting in a dataset of 203 loci for 345 individuals with a mean genotyping error rate of 4.1%.

### 4.3. Population Structure

The hierarchical structure in the AFLP dataset was investigated using the Bayesian genotype clustering method adapted to dominant markers, implemented in the program STRUCTURE v. 2.3.4 [65,66]. The program allows estimation of the most likely number of genetic clusters in a particular dataset by grouping individuals such that Hardy-Weinberg equilibrium is maximized within clusters. The optimal number of genetic clusters *K* was determined using the modal distribution of the ∆K statistic of Evanno et al. [48] with STRUCTURE HARVESTER v. 0.6.94 [67]. In datasets with complex structures, the Evanno approach tends to detect the uppermost level of hierarchical structure (i.e., the main divergent groups), but not necessarily the optimal number of populations. As our dataset is composed of potentially fully differentiated populations (species), after the first STRUCTURE analysis that suggested the existence of dichotomic clusters (*K* = 2), the dataset was subset according to the results of the individual assignments for *K* = 2 and analyses were repeated for each cluster independently. For the analysis of the complete dataset, *K* = 1–16 was used, while analyses on the subsets were performed for *K* = 1–12 and *K* = 1–8 based on the estimated number of populations. For each type of analysis and each *K* value, three replicate chains of 175,000 MCMC iterations were run and the first 25,000 iterations were discarded as a burn-in. STRUCTURE was used with the default settings except for the application of the recessive allele option and the admixture model with correlated allele frequencies. The average membership coefficients for the three simulation runs of a given *K* value were generated with CLUMPAK v. 1.1 [68].

### 4.4. Descriptive Statistics

Allele frequencies were estimated for each population from AFLP fragment frequencies with the Bayesian method of Zhivotovsky [69], implemented in the software AFLPsurv v. 1.0 [70], using a non-uniform prior distribution and assuming that populations were at the Hardy-Weinberg equilibrium. The resulting allele frequencies were used to estimate the expected heterozygosity or Nei’s gene diversity (*H_j_*) [71] for each population and to compute the genetic differentiation across all populations (F_st_) using 1000 permutations. A thousand matrices of Nei’s unbiased genetic distances were computed by bootstrapping over AFLP loci in AFLPsurv and exported to PHYLIP software package v. 3.6 [72] to build a neighbor-joining (NJ) dendrogram with bootstrap support on branches. To compare the portioning of the genetic variation between our a priori species hypothesis and the clusters identified at the issue of the two steps clustering analyses with STRUCTURE, two hierarchical analyses of molecular variance (AMOVA) were performed in Arlequin v. 3.5.1.3 [73]. Populations were first grouped by species, then by genetic clusters (see Table 1).

### 4.5. Spatial Genetic Structure

To estimate the spatial genetic structure (SGS) in the main genetic clusters identified by STRUCTURE, the pairwise kinship coefficient was calculated with the software SPAGeDi v1.5.a [46]. The estimator for the kinship coefficient for dominant genetic markers developed by Hardy [74] requires the user to specify an estimate of the inbreeding coefficient. Here, we assumed a null inbreeding coefficient. Then, kinship coefficients were associated with paired spatial distance in correlograms with 9 distance classes for the cluster composed of *M. xerantifulva* and *M. claireae* (0.1, 0.5, 2, 5, 10, 20, 50, 100, 200 km) and with 11 distances classes for the cluster composed by the other species (0.1, 0.5, 2, 5, 10, 20, 50, 100, 200, 400, 800 km). These relatively large distance classes, compared with what is found in the literature, were selected to accommodate our sparse sampling design. While it does not allow us to determine the fine SGS, it will already indicate how genetic structure is influenced by distance. The significance of the average kinship coefficient in every distance class was tested using 1000 permutations of spatial locations and individuals, while standard errors were estimated by Jackknifing across loci. The permutation procedure also allowed testing of whether the regression slope of the kinship coefficient over the distance was significantly negative, as expected under a scenario of isolation by distance. Both the kinship coefficient and the regression slopes are affected by the sampling design and thus are not appropriate for comparison between species. To compare the strength of SGS between genetic clusters, the *Sp* statistic [53] was computed as *Sp* = *−b*_F_/(1 − *F*_(1)_), where *b*_F_ is the slope of the regression of kinship against ln distance and *F*_(1)_ is the average kinship of individuals in the first distance class (here 0–100 m). Because this statistic is calculated on the logarithm of the distance it is less affected by the sampling design and thus has the desirable advantage of being comparable across studies.

## 5. Conclusions

We showed that the species delimitation in this young *Macrocarpaea* species complex composed of several morphological cryptic species requires new consideration. However, the highly asymmetrical sampling in terms of the number of individuals and populations per species, as well as the relatively small AFLP dataset included in this study, does not allow unambiguous estimation regarding their genetic structure, phylogeny, evolution history and prevailing evolutionary processes. More extensive sampling with even representation of all populations and application of modern genetic methods (such as next-generation sequencing) and corresponding analyses more suitable for population genetics, evolutionary reconstructions and related questions are required for more precise conclusions. This is especially valid for the genetically complex cluster B that regroups the essential of the supposed taxonomic diversity in this species complex.

Nevertheless, we were able to demonstrate that *M. claireae* and *M. xerantifulva* may form a single species that evolved in relative isolation from the rest of the species complex. Relying on denser sampling, we show that this species has relatively low gene diversity that could have resulted from a recent demographic bottleneck. We suggest that the intensification of drought induced by the PCO in the Rio Chinchipe valley might have compressed this species in refugia. While remaining highly speculative, this statement suggests that the different Andean valleys might have responded in a dramatically different way to the PCO. The easternmost valleys remain wet while the more internal ones, which are currently in contact with dry systems such as the Rio Marañón, suffer from drought. However, it would be highly desirable in the future, with the application of more modern methods and extensive sampling, to perform a reevaluation of the effect of the Pleistocene climatic oscillations compared to other potential evolutionary processes, as well as mountain geomorphology and climatic and potential other influential factors to confirm or correct our findings.

## 6. Simple Summary

The effect of the Pleistocene climatic oscillations on species evolution in the neotropical mountains is poorly known. Here, we used amplified fragment length polymorphism (AFLPs) genetic markers to analyze the phylogeography and population genetics of several lineages belonging to the plant genus *Macrocarpaea* (Gentianaceae) occurring in the tropical Andes. We test which climatic scenario was the most plausible during the Pleistocene glacial periods and what impact the abiotic changes had on montane forest populations.

## Figures and Tables

**Table 1:** List of populations included in this study. For each population, a voucher ID, country of origin (E: Ecuador, P: Peru), region of origin (C: Carchi, EO: El Oro, J: Jaén, MS: Morona-Santiago, P: Pichincha, S: Sucumbíos, SA: San Ignacio, SM: San Martin, ZC: Zamora-Chinchipe), main genetic clusters (A and B) and sub-clusters (1 to 4) identified by STRUCTURE, mean geographical coordinates (calculated as means for all samples in the given population), mean altitude (in meters above sea level, calculated as means for all samples in the given population), sample size N, the proportion of polymorphic markers (PLP), the gene diversity (*H_j_*) and standard error of the *H_j_* (S.E. (*H_j_*)) are provided.

Population	Voucher	Country	Region	Cluster	Latitude	Longitude	Altitude	N	PLP	*H_j_*	S.E. (*H_j_*)
*M. claireae* 1	JRG4666	E	ZC	A1	−4.5422	−79.1304	1744	19	21.7	0.090	0.009
*M. claireae* 2	JRG4667	E	ZC	A1	−4.5590	−79.1371	1571	17	39.9	0.116	0.011
*M. cortinae* 1 *M. dies-viridis* 1	JRG5114–5116 JRG4693	E E	S ZC	B1 B3	0.3671 −3.9009	−77.4923 −78.5117	1562 1609	12 9	38.9 43.8	0.136 0.119	0.011 0.011
*M. dies-viridis* 2	JRG4679	E	ZC	B3	−3.9373	−78.6245	1353	10	48.3	0.143	0.012
*M. dies-viridis* 3	JRG4679	E	ZC	B3	−3.9263	−78.6235	1545	20	49.8	0.136	0.011
*M. dies-viridis* 4	JRG4693	E	ZC	B3	−3.9120	−78.5040	1742	6	44.3	0.127	0.011
*M. illuminata* 1	JRG4687	E	ZC	B1	−3.9673	−78.6863	912	10	54.7	0.157	0.012
*M. illuminata* 2	JRG4679	E	ZC	B1	−3.9378	−78.7200	985	7	41.9	0.139	0.012
*M.* sp. nov.	JRG4700	E	ZC	B1	−3.5363	−78.5288	777	14	44.8	0.152	0.011
*M. lenae*	JRG4675	E	ZC	B2	−4.0891	−78.9653	1110	18	56.2	0.168	0.011
*M. pacifica* 1	JRG4704	E	EO	B2	−3.6509	−79.7469	934	11	58.6	0.162	0.012
*M. pringleana* 1	JRG5118, -20, -21	E	MS	B1	−2.2073	−78.1985	1305	5	37.4	0.128	0.012
*M. quizhpei* 1	JRG4688	E	ZC	B2	−4.1809	−78.6441	863	13	44.8	0.162	0.013
*M. quizhpei* 2	JRG4689	E	ZC	B2	−4.2503	−78.6613	933	18	52.2	0.180	0.013
*M. quizhpei* 3	JRG4688	E	ZC	B2	−4.2597	−78.6481	1026	5	45.8	0.191	0.014
*M. quechua* 1	JV20	P	SM	B1	−6.4560	−76.2914	675	7	39.9	0.122	0.011
*M. sodiroana* 1	JRG5103, -06	E	P	B1	0.3033	−78.8689	1453	8	43.3	0.125	0.011
*M. umbellata* 1	JRG5107	E	C	B1	0.9156	−78.2013	1497	2	36.9	0.207	0.015
*M. xerantifulva* 1	JV27	P	J	A3	−5.9771	−79.0516	1867	4	3.9	0.032	0.005
*M. xerantifulva* 2	JV26	P	J	A2	−5.6830	−78.8715	1887	2	34	0.116	0.011
*M. xerantifulva* 3	JV26	P	J	A3	−5.6777	−78.8779	2039	5	8.9	0.061	0.008
*M. xerantifulva* 5	JV26	P	J	A3	−5.6840	−78.9044	1758	8	36.9	0.079	0.009
*M. xerantifulva* 6	JV26	P	J	A3	−5.6824	−78.9032	1827	17	35	0.112	0.012
*M. xerantifulva* 7	JV26	P	J	A3	−5.6754	−78.9116	1858	6	36.9	0.099	0.010
*M. xerantifulva* 8	JV46bis	P	J	A3	−5.6857	−78.9328	1943	5	32.5	0.088	0.010
*M. xerantifulva* 9	JV46	P	J	A4	−5.7051	−78.9443	2085	3	32	0.099	0.010
*M. xerantifulva* 10	JV46	P	J	A3	−5.7023	−78.9335	1859	3	3.4	0.036	0.005
*M. xerantifulva* 11	JV44	P	J	A3	−5.5770	−78.8976	1714	10	42.4	0.107	0.011
*M. xerantifulva* 12	JV44	P	J	A3	−5.5816	−78.9428	1908	3	34.5	0.132	0.012
*M. xerantifulva* 13	JV44	P	J	A3	−5.5788	−78.9494	1795	14	11.3	0.060	0.008
*M. xerantifulva* 14	JV44	P	J	A3	−5.5748	−78.9710	1549	5	34.5	0.086	0.010
*M. xerantifulva* 15	JV44	P	J	A3	−5.5655	−78.9730	1588	5	37.4	0.089	0.009
*M. xerantifulva* 16	JV28	P	J	A2	−5.3705	−78.9104	1648	17	41.4	0.105	0.010
*M. xerantifulva* 17	JV28	P	J	A3	−5.3747	−78.9378	1760	12	35.5	0.082	0.009
*M. xerantifulva* 18 *M. xerantifulva* 19	JV29 JRG4666bis	P E	SI ZC	A2 A2	−5.1384 −4.8490	−79.0337 −79.2464	1472 1581	13 2	41.9 32.5	0.121 0.073	0.010 0.009

**Table 2:** Summary of the hierarchical analyses of molecular variance (AMOVA) performed in Arlequin by grouping: (1) the populations by species and (2) by genetic clusters identified by the two-step STRUCTURE analyses. *p*-values were obtained with 1023 permutations. F_CT_ = variation among groups divided by total variation, F_SC_ = variation among sub-groups within groups divided by the sum of variation among sub-groups within groups and variation within sub-groups, F_ST_ = the sum of variation among groups and variation among sub-groups within groups, divided by total variation.

Source of Variation	Df	Variance Components	Percentage of Variation	*F* Statistics	*p*-Value
Among species	10	6.25	33.06	F_CT_ 0.33	<0.001
Among populations/species	26	2.28	12.06	F_SC_ 0.18	<0.001
Within populations	308	10.38	54.88	F_ST_ 0.45	<0.001
Total	344	18.91			
Among clusters	6	4.96	27.25	F_CT_ 0.27	<0.001
Among populations/clusters	30	2.85	15.68	F_SC_ 0.22	<0.001
Within populations	308	10.38	57.07	F_ST_ 0.43	<0.001
Total	344	18.19			

## Data Availability

Additional datasets generated and analyzed in this study that are not presented here or in the Supplementary Materials are available upon request from the corresponding author.

## Supplementary Materials

The following are available online at https://www.mdpi.com/article/10.3390/plants12081710/s1, Figure S1: Outputs of the STRUCTURE analyses. On the left side—plots of the ∆*K* statistic of Evanno et al. (2005) for detecting the number of K groups that best fit the data. On the right side—the plot of mean likelihood L(*K*) and variance per *K* value from STRUCTURE. (A) Analyses performed including all the individuals. (B) Analyses performed including the population assigned to the genetic cluster A. (C) Analyses performed on the populations assigned to the genetic cluster B.

## Abbreviations

a.s.l.	above sea level
AHZ	Amotape-Huancabamba zone
AFLP	amplified fragment length polymorphism genetic markers
AMOVA	analysis of molecular variance
LMF	lower montane forest
MMF	middle elevation montane forest
MRCA	most recent common ancestor
mya	million years ago
Myr	million years
NJ	neighbor-joining
PCO	Pleistocene climatic oscillations
SDTF	Seasonally dry tropical forests
SGS	spatial genetic structure
UFL	upper forest line
UMF	upper montane forest

## References

[1] Afzan A., Bréant L., Bellstedt D.U., Grant J.R., Queiroz E.F., Wolfender J.-L., Kissling J. (2019). Can biochemical phenotype, obtained from herbarium samples, help taxonomic decisions?—A case study using Gentianaceae. TAXON.

[2] Baker P.A., Bush M., Fritz S., Rigsby C.A., Seltzer G., Silman M. (2004). Last Glacial Maximum in an Andean cloud forest environment (Eastern Cordillera, Bolivia): Comment and Reply: COMMENT. Geology.

[3] Bonin A., Bellemain E., Bronken Eidesen P., Pompanon F., Brochmann C., Taberlet P. (2004). How to track and assess genotyping errors in population genetics studies. Mol. Ecol..

[4] Brunschön C., Behling H. (2010). Reconstruction and visualization of upper forest line and vegetation changes in the Andean depression region of southeastern Ecuador since the last glacial maximum—A multi-site synthesis. Rev. Palaeobot. Palynol..

[5] Bush M.B., de Oliveira P.E. (2006). The rise and fall of the Refugial Hypothesis of Amazonian speciation: A paleoecological perspective. Biota Neotrop..

[6] Colinvaux P.A., De Oliveira P.E., Bush M.B. (2000). Amazonian and neotropical plant communities on glacial time-scales: The failure of the aridity and refuge hypotheses. Quat. Sci. Rev..

[7] Collevatti R., Lima J.S., Soares T., Telles M. (2010). Spatial genetic structure and life history traits in cerrado tree species: Inferences for conservation. Nat. Conserv..

[8] Coulon A., Fitzpatrick J.W., Bowman R., Stith B.M., Makarewich C.A., Stenzler L.M., Lovette I.J. (2008). Congruent population structure inferred from dispersal behaviour and intensive genetic surveys of the threatened Florida scrub-jay (*Aphelocoma coerulescens*). Mol. Ecol..

[9] Crespi E.J., Rissler L.J., Browne R.A. (2003). Testing Pleistocene refugia theory: Phylogeographical analysis of *Desmognathus wrighti*, a high-elevation salamander in the southern Appalachians. Mol. Ecol..

[10] Dlugosch K.M., Parker I.M. (2008). Founding events in species invasions: Genetic variation, adaptive evolution, and the role of multiple introductions. Mol. Ecol..

[11] Durand E., Jay F., Gaggiotti O.E., François O. (2009). Spatial inference of admixture proportions and secondary contact zones. Mol. Biol. Evol..

[12] Earl D.A., von Holdt B.M. (2012). STRUCTURE HARVESTER: A website and program for visualizing STRUCTURE output and implementing the Evanno method. Conserv. Genet. Resour..

[13] Elias M., Joron M., Willmott K., Silva-Brandão K.L., Kaiser V., Arias C.F., Gomez Piñerez L.M., Uribe S., Brower A.V.Z., Freitas A.V.L. (2009). Out of the Andes: Patterns of diversification in clearwing butterflies. Mol. Ecol..

[14] Evanno G., Regnaut S., Goudet J. (2005). Detecting the number of clusters of individuals using the software STRUCTURE: A simulation study. Mol. Ecol..

[15] Excoffier L., Lischer H.E.L. (2010). Arlequin suite ver 3.5: A new series of programs to perform population genetics analyses under Linux and Windows. Mol. Ecol. Resour..

[16] Falush D., Stephens M., Pritchard J.K. (2003). Inference of population structure using multilocus genotype data: Linked loci and correlated allele frequencies. Genetics.

[17] Felsenstein J. (2005). PHYLIP (Phylogeny Inference Package) Version 3.6, Distributed by the Author.

[18] Fjeldså J., Bowie R.C.K., Rahbek C. (2012). The role of mountain ranges in the diversification of birds. Annu. Rev. Ecol. Evol. Syst..

[19] Flantua S.G.A., Hooghiemstra H., van Boxel J.H., Cabrera M., González-Carranza Z., González-Arango C. (2014). Connectivity Dynamics since the Last Glacial Maximum in the Northern Andes: A Pollen-Driven Framework to Assess Potential Migration.

[20] Fleming T.H., Geiselman C., Kress W.J. (2009). The evolution of bat pollination: A phylogenetic perspective. Ann. Bot..

[21] Gentry A.H. (1982). Neotropical floristic diversity: Phytogeographical connections between Central and South America, Pleistocene climatic fluctuations, or an accident of the Andean ogrogeny?. Ann. Mo. Bot. Gard..

[22] Grant J.R., Rybczyński J.J., Davey M.R., Mikuła A. (2014a). A monographic revision of the neotropical genus Macrocarpaea (Gentianaceae) in Ecuador. The Gentianaceae—Volume 1: Characterization and Ecology.

[23] Grant J.R. (2014b). *De Macrocarpaeae Grisebach (Ex Gentianaceis) Speciebus novis* XI: Five new species from the Andes of Ecuador and Colombia. Harvard Pap. Bot..

[24] Grant J.R. (2011). *De Macrocarpaeae Grisebach (ex Gentianaceis) Speciebus novis* IX: A Synopsis of the Genus in Bolivia. Harvard Pap. Bot..

[25] Grant J.R. (2008). *De Macrocarpaeae Grisebach (ex Gentianaceis) Speciebus novis* VIII: Two new species from Ecuador. Harvard Pap. Bot..

[26] Grant J.R. (2007). *De Macrocarpaeae Grisebach (ex Gentianaceis) Speciebus novis* VII: Four new species and two natural hybrids. Harvard Pap. Bot..

[27] Grant J.R. (2005). *De Macrocarpaeae Grisebach (ex Gentianaceis) Speciebus novis* VI: Seed morphology, palynology, an infrageneric classification, and another twenty-three new species, largely from Colombia. Harvard Pap. Bot..

[28] Grant J.R. (2004). *De Macrocarpaeae Grisebach (ex Gentianaceis) Speciebus novis* V: Twenty-three new species largely from Peru, and typification of all species in the genus. Harvard Pap. Bot..

[29] Grant J.R. (2003). *De Macrocarpaeae Grisebach (ex Gentianaceis) Speciebus Novis* II: Typification of the Ruiz & Pavon names. Harvard Pap. Bot..

[30] Grant J.R., Struwe L. (2003). *De Macrocarpaeae Grisebach (ex Gentianaceis) Speciebus novis* III: Six new species of moon-gentians (*Macrocarpaea*, Gentianaceae: Helieae) from Parque Nacional Podocarpus, Ecuador. Harvard Pap. Bot..

[31] Grant J.R., Struwe L. (2001). *De Macrocarpaeae Grisebach (ex Gentianaceis) Speciebus Novis* I: An introduction to the genus *Macrocarpaea* and three new species from Colombia, Ecuador, and Guyana. Harvard Pap. Bot..

[32] Grant J.R., Trunz V. (2011). *De Macrocarpaeae Grisebach (ex Gentianaceis) Speciebus novis* X: A synopsis of the genus in Montane Atlantic Forests of Brazil. Harvard Pap. Bot..

[33] Grant J.R., Vieu J. (2014). *De Macrocarpaeae Grisebach (ex Gentianaceis) Speciebus novis* XII: Three new species from the Andes of Peru. Harvard Pap. Bot..

[34] Grant J.R., Weaver R.E. (2003). *De Macrocarpaeae Grisebach (ex Gentianaceis) Speciebus novis* IV: Twelve new species of *Macrocarpaea* (Gentianaceae: Helieae) from Central and South America, and the first report of the presence of a stipule in the family. Harvard Pap. Bot..

[35] Hardy O.J. (2003). Estimation of pairwise relatedness between individuals and characterization of isolation-by-distance processes using dominant genetic markers. Mol. Ecol..

[36] Hardy O.J., Maggia L., Bandou E., Breyne P., Caron H., Chevallier M.-H., Doligez A., Dutech C., Kremer A., Latouche-Hallé C. (2006). Fine-scale genetic structure and gene dispersal inferences in 10 neotropical tree species. Mol. Ecol..

[37] Hardy O.J., Vekemans X. (2002). SPAGeDi: A versatile computer program to analyse spatial genetic structure at the individual or population levels. Mol. Ecol. Notes.

[38] Herrmann D., Poncet B.N., Manel S., Rioux D., Gielly L., Taberlet P., Gugerli F. (2010). Selection criteria for scoring amplified fragment length polymorphisms (AFLPs) positively affect the reliability of population genetic parameter estimates. Genome.

[39] Hewitt G.M. (2004). Genetic consequences of climatic oscillations in the Quaternary. Philos. Trans. R. Soc. London. Ser. B Biol. Sci..

[40] Hewitt G.M. (1996). Some genetic consequences of ice ages, and their role in divergence and speciation. Biol. J. Linn. Soc..

[41] Hughes C., Eastwood R. (2006). Island radiation on a continental scale: Exceptional rates of plant diversification after uplift of the Andes. PNAS.

[42] Hughes C.E., Atchison G.W. (2015). The ubiquity of alpine plant radiations: From the Andes to the Hengduan Mountains. New Phytol..

[43] Hutter C.R., Guayasamin J.M., Wiens J.J. (2013). Explaining Andean megadiversity: The evolutionary and ecological causes of glassfrog elevational richness patterns. Ecol. Lett..

[44] Knowles L.L. (2000). Tests of Pleistocene speciation in montane grasshoppers (genus *Melanoplus*) from the Sky Islands of western North America. Evolution.

[45] Kopelman N.M., Mayzel J., Jakobsson M., Rosenberg N.A., Mayrose I. (2015). Clumpak: A program for identifying clustering modes packaging population structure inferences across, K. Mol. Ecol. Resour..

[46] Linder H.P., Rabosky D.L., Antonelli A., Wüest R.O., Ohlemüller R. (2014). Disentangling the influence of climatic and geological changes on species radiations. J. Biogeogr..

[47] Lynch M., Milligan B.G. (1994). Analysis of population genetic structure with RAPD markers. Mol. Ecol..

[48] Moritz C., Patton J.L., Schneider C.J., Smith T.B. (2000). Diversification of rainforest faunas: An integrated molecular approach. Annu. Rev. Ecol. Syst..

[49] Mourguiart P., Ledru M.-P. (2003). Last Glacial Maximum in an Andean cloud forest environment (Eastern Cordillera, Bolivia). Geology.

[50] Murray M.G., Thompson W.F. (1980). Rapid isolation of high molecular weight plant, D.N.A. Nucleic Acids Res..

[51] Nybom H. (2004). Comparison of different nuclear DNA markers for estimating intraspecific genetic diversity in plants. Mol. Ecol..

[52] Orme C.D.L., Davies R.G., Burgess M., Eigenbrod F., Pickup N., Olson V.A., Webster A.J., Ding T.-S., Rasmussen P.C., Ridgely R.S. (2005). Global hotspots of species richness are not congruent with endemism or threat. Nature.

[53] Parisod C., Christin P.-A. (2008). Genome-wide association to fine-scale ecological heterogeneity within a continuous population of *Biscutella laevigata* (Brassicaceae). New Phytol..

[54] Pritchard J.K., Stephens M., Donnelly P. (2000). Inference of population structure using multilocus genotype data. Genetics.

[55] Qin A., Ding Y., Jian Z., Ma F., Worth J.R.P., Pei S., Xu G., Guo Q., Shi Z. (2021). Low genetic diversity and population differentiation in Thuja sutchuenensis Franch., an extremely endangered rediscovered conifer species in southwestern China. Glob. Ecol. Conserv..

[56] Ramírez-Barahona S., Eguiarte L.E. (2013). The role of glacial cycles in promoting genetic diversity in the Neotropics: The case of cloud forests during the Last Glacial Maximum. Ecol. Evol..

[57] Rull V. (2008). Speciation timing and neotropical biodiversity: The Tertiary-Quaternary debate in the light of molecular phylogenetic evidence. Mol. Ecol..

[58] Schmitt T. (2007). Molecular biogeography of Europe: Pleistocene cycles and postglacial trends. Front. Zool..

[59] Schönswetter P., Stehlik I., Holderegger R., Tribsch A. (2005). Molecular evidence for glacial refugia of mountain plants in the European Alps. Mol. Ecol..

[60] Schwery O., Onstein R.E., Bouchenak-Khelladi Y., Xing Y., Carter R.J., Linder H.P. (2015). As old as the mountains: The radiations of the Ericaceae. New Phytol..

[61] Smith S.A., Oca A.N.M.D., Reeder T.W., Wiens J.J. (2007). A phylogenetic perspective on elevational species richness patterns in middle American treefrogs: Why so few species in lowland tropical rainforests?. Evolution.

[62] Struwe L., Albert V.A., Calió F.M., Frasier C., Lepis K.B., Mathews K.G., Grant J.R. (2009a). Evolutionary patterns in neotropical Helieae (Gentianaceae): Evidence from morphology, chloroplast and nuclear DNA sequences. TAXON.

[63] Struwe L., Haag S., Heiberg E., Grant J.R. (2009b). Andean speciation and vicariance in neotropical *Macrocarpaea* (Gentianaceae-Helieae). Ann. Mo. Bot. Gard..

[64] Trénel P., Hansen M.M., Normand S., Borchsenius F. (2008). Landscape genetics, historical isolation and cross-Andean gene flow in the wax palm, *Ceroxylon echinulatum* (Arecaceae). Mol. Ecol..

[65] van der Hammen T., Hooghiemstra H. (2000). Neogene and Quaternary history of vegetation, climate, and plant diversity in Amazonia. Quat. Sci. Rev..

[66] Vekemans X., Beauwens T., Lemaire M., Roldán-Ruiz I. (2002). Data from amplified fragment length polymorphism (AFLP) markers show indication of size homoplasy and of a relationship between degree of homoplasy and fragment size. Mol. Ecol..

[67] Vekemans X., Hardy O.J. (2004). New insights from fine-scale spatial genetic structure analyses in plant populations. Mol. Ecol..

[68] Vieu J.C., Hughes C.E., Kissling J., Grant J.R. (2022). Evolutionary diversification in the hyper-diverse montane forests of the tropical Andes: Radiation of Macrocarpaea (Gentianaceae) and the possible role of range expansion. Bot. J. Linn. Soc..

[69] Vieu J.C., Koubínová D., Grant J.R. (2021). The Evolution of Trait Disparity during the Radiation of the Plant Genus Macrocarpaea (Gentianaceae) in the Tropical Andes. Biology.

[70] Vos P., Hogers R., Bleeker M., Reijans M., van de Lee T., Hornes M., Frijters A., Pot J., Peleman J., Kuiper M. (1995). AFLP: A new technique for DNA fingerprinting. Nucleic Acids Res..

[71] Wolff D. (2006). Nectar sugar composition and volumes of 47 species of Gentianales from a southern Ecuadorian montane forest. Ann. Bot..

[72] Wollenberg K.C., Vieites D.R., Meijden A.V.D., Glaw F., Cannatella D.C., Vences M. (2008). Patterns of endemism and species richness in Malagasy cophyline frogs support a key role of mountainous areas for speciation. Evolution.

[73] Zhang X., Yang L., Liu Y.-H., Zhou X.-L., Zhang L.-Q., Wang Y.-H., Shen S.-K. (2021). Genetic diversity, genetic structure, and demographic history of Cinnamomum chago, a plant species with extremely small populations in China. Glob. Ecol. Conserv..

[74] Zhivotovsky L.A. (1999). Estimating population structure in diploids with multilocus dominant DNA markers. Mol. Ecol..

