# Supplementary material for: Population Genetic Structure and Diversity of Cryptic Species of the Plant Genus Macrocarpaea (Gentianaceae) from the Tropical Andes
Source: Plants (Basel). 2023 Apr 20;12(8):1710. doi: 10.3390/plants12081710 (PMC10145315; doi:10.3390/plants12081710)
Supplement: Supplementary file 1 [file plants-12-01710-s001.zip › plants-2251873-supplementary.pdf]

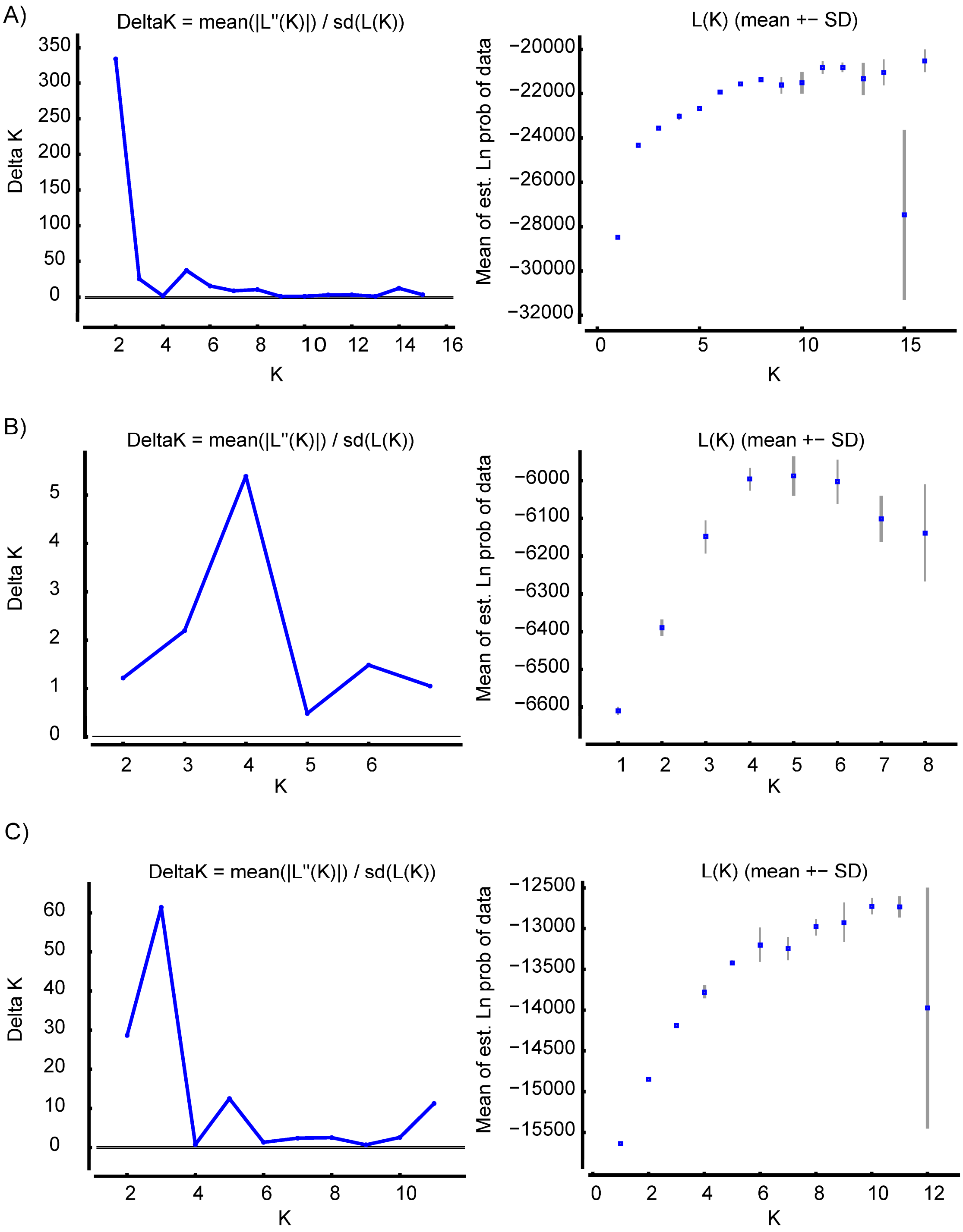

Figure S1. Outputs of the STRUCTURE analyses. On the left side—plots of the Δ*K* statistic of Evanno et al. (2005) for detecting the number of K groups that best fit the data. On the right side—the plot of mean likelihood L(*K*) and variance per *K* value from STRUCTURE. (**A**) Analyses performed including all the individuals. (**B**) Analyses performed including the population assigned to the genetic cluster A. (**C**) Analyses performed on the populations assigned to the genetic cluster B.
